# A Case Report and Literature Review of a Rare Jejunal Solitary Peutz–Jeghers-Type Polyp Resected Endoscopically in an Elderly Patient Presenting with Subacute Gastrointestinal Bleeding

**DOI:** 10.1155/2023/2391602

**Published:** 2023-12-18

**Authors:** Talal Alenezi, Victoria Marcus, Talat Bessissow

**Affiliations:** ^1^Division of Gastroenterology and Hepatology, McGill University Health Centre, Montreal, QC, Canada; ^2^Department of Pathology, McGill University Health Centre, Montreal, QC, Canada

## Abstract

Solitary Peutz–Jeghers-type polyp (SPJP) is a rare hamartomatous lesion. It is considered a different entity from Peutz–Jeghers syndrome despite similar histopathological findings. It can be found in the GI tract but rarely in the jejunum. Jejunal SPJP is susceptible to necrosis, ulceration, and intussusception, resulting in GI bleeding or small bowel obstruction. We describe a case of subacute gastrointestinal bleeding secondary to jejunal SPJP to share our approach to this challenging case using therapeutic endoscopy. An 81-year-old male patient with a history of atrial fibrillation on warfarin with stable therapeutic INR levels presented with a 1-week history of melena, generalized fatigue, and shortness of breath on exertion and was found to have profound iron deficiency anemia. Esophageal gastroduodenoscopy and colonoscopy failed to identify the source of bleeding; however, single-balloon enteroscopy detected a 4 cm polyp with a stalk in the proximal jejunum. Endoscopic polypectomy was performed, and the whole polyp was removed. Histopathological examination was consistent with Peutz–Jeghers polyp. The genetic analysis was negative for STK11 mutation. Follow-up magnetic resonance enterography and video capsule endoscopy did not reveal any other polypoid lesion in the GI tract. The patient's symptoms resolved gradually, and his hemoglobin level returned back to normal levels within 6 months. To our knowledge, this is the first case of endoscopic polypectomy during balloon-assisted enteroscopy for jejunal SPJP.

## 1. Introduction

Solitary Peutz–Jeghers-type polyp (SPJP) is a rare hamartomatous lesion, characterized by the absence of mucocutaneous hyperpigmentation and a family history of Peutz–Jeghers syndrome [1]. It is described histopathologically by bundles of interdigitating smooth muscles throughout the lamina propria, giving the appearance of a branching tree. Compared to other hamartomas with polyposis syndromes, small bowel SPJP is less commonly encountered in clinical practice [2]. Genetically, it is distinct from Peutz–Jeghers syndrome as it lacks somatic and germline mutations at the STK11 locus [3]. Despite the genetic differences, the histopathological features and the clinical presentation appear to be similar to other small bowel hamartomas [2]. Symptomatic patients with small bowel SPJP can present with different gastrointestinal manifestations, including abdominal pain, vomiting, and gastrointestinal bleeding. Jejunal hamartomas are susceptible to necrosis, ulceration, and intussusception, resulting in gastrointestinal (GI) bleeding or obstruction. We described a case of subacute GI bleeding secondary to a jejunal SPJP to share our approach to this challenging case using balloon-assisted enteroscopy.

## 2. Case Presentation

**Figure 1:**
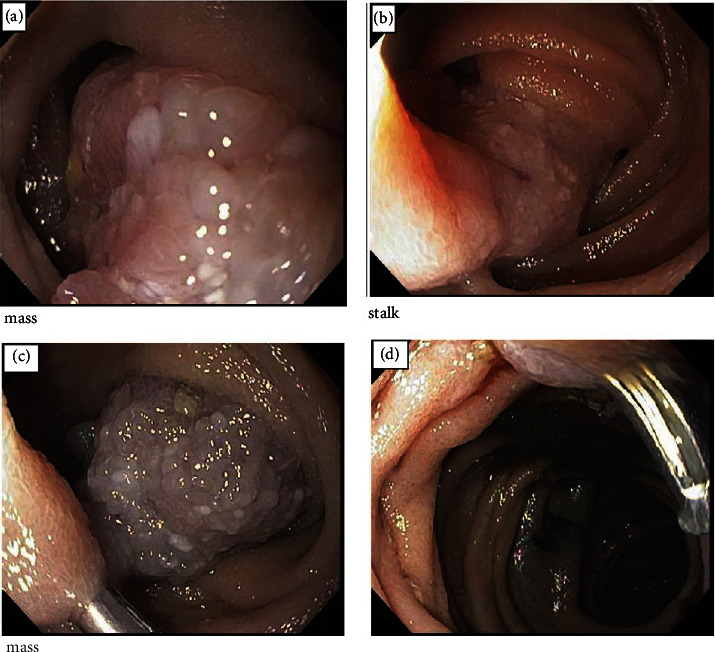
(a) Jejunal polyp, (b) stalk, (c) metallic clip placed on the stalk, and (d) postpolypectomy site and tattoo injection.

**Figure 2:**
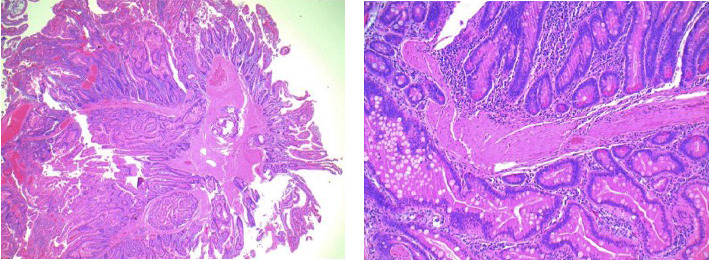
Histological appearance of the Peutz–Jeghers hamartomatous polyp. (a) Low-power (20× magnification, hematoxylin and eosin (H&E)) and (b) medium-power (100×, H&E) images of Peutz–Jeghers hamartomatous polyp showing smooth muscle tree-like branching (so-called “arborization”) with overlying nondysplastic small intestinal epithelium.

An 81-year-old gentleman presented to the internal medicine clinic with a 1-week history of melena, generalized fatigue, and shortness of breath on exertion. His past medical history includes atrial fibrillation CHADs65 score of 1, on warfarin with stable therapeutic international ratio (INR) levels. He is also known to have gout and benign prostatic hyperplasia but no other medical comorbidities. He had a remote partial esophagectomy for a leiomyoma of the esophagus and a remote appendectomy. Colonoscopy 4 years prior to this presentation was normal except for a small sessile serrated adenomatous polyp at the ileocecal valve. His medication list included warfarin, bisoprolol, tamsulosin, and finasteride. There was no recent use of NSAIDs or antiplatelets. His family history was negative for gastrointestinal diseases, or malignancies, and his social history was not contributory to this presentation. There was no associated nausea, vomiting, or abdominal discomfort. He denied having abdominal pain, constipation, or diarrhea. His history was negative for hematemesis and bright red blood per rectum. The abdominal examination was unremarkable, and the rest of the physical examination was normal. Blood investigations revealed microcytic hypochromic anemia and a subacute drop of Hgb level to 10.8 g/dL from a baseline of 16.0 g/dL 8 months earlier. The INR was in the therapeutic range, and platelet levels were normal. The iron profile was consistent with iron deficiency anemia, and the gastroenterology team was consulted for further evaluation. Esophageal gastroduodenoscopy (EGD) and colonoscopy failed to identify the source of bleeding. The patient underwent a single-balloon enteroscopy that detected a solitary large pedunculated polypoid lesion with a stalk in the proximal jejunum about 4-5 cm in size (Figure 1). A prophylactic metallic clip was placed on the stalk, and endoscopic polypectomy was performed using a snare cautery technique. The whole polyp was removed, and a tattoo was injected beside the base of the polyp. Histopathological evaluation of the resected sample was consistent with Peutz–Jeghers polyp (Figure 2). A follow-up magnetic resonance enterography and a video capsule endoscopy were negative for other polypoid lesions in the gastrointestinal tract, and the genetic testing was negative for STK11 mutation. The patient was started on ferrous fumarate oral tablets. His symptoms improved gradually and Hgb level returned back to his baseline within 6 months.

## 3. Discussion

Solitary Peutz–Jegher polyp in the jejunum is a rare clinical finding. The first reported case in English literature was described by Sone et al. in 2000 [4]; polypectomy was performed during an upper GI endoscopy in a patient presenting with abdominal pain. That patient had distal gastrectomy with Billroth II reconstruction for gastric cancer. Since then, additional 7 case reports have been published, 3 in pediatric and 4 in adult patients aged >18 years, all required surgical intervention (Table 1). Six cases presented with intussusception and only 1 patient presented with melena and GI bleeding. To our knowledge, this is the 2^nd^ case of GI bleeding unrelated to intussusception from jejunal SPJP, and the first case to be treated endoscopically during a single-balloon enteroscopic evaluation.

Most cases of small bowel hamartomas present as surgical emergencies with small bowel obstructions, intussusception, or GI bleeding requiring urgent surgical intervention. A systematic review of 39 cases with different types of symptomatic ileal and jejunal hamartomas highlighted the common clinical presentations. Abdominal pain was the most common symptom, and intussusception was the most common underlying pathophysiology. Eight patients presented with GI bleeding as the initial symptoms, and the bleeding occurred secondary to ulceration or necrosis of the hamartomas in 5 of these cases [12]. Furthermore, the systematic review highlighted the different therapeutic modalities for each presentation. All of the 39 cases underwent surgical resection eventually [12].

In this report, the mechanism of bleeding was not clear. Although intussusception is known to cause GI bleeding, the patient in our study did not report any symptoms to suggest it. Ulceration and necrosis of the hamartoma is a possible mechanism; however, the morphological appearance of the resected polypoid lesion was not consistent with that. Despite the uncertainty about the bleeding mechanism in this case, the clinical consequences of jejunal SPJP are comparable to other small bowel hamartomas.

Individuals with Peutz–Jeghers syndrome are at increased risk of different types of malignancies, such as gynecological, GI, and pancreaticobiliary carcinomas [13]. On the other hand, the malignant potential of SPJP is controversial [14]. Initial studies in the early 2000s suggested a benign course [15]; however, subsequent studies have contradicted the initial thoughts. There have been cases that reported an association between SPJS with different malignancies [14]. Another study looked at the genetic and epigenetic analysis of colonic SPJP and detected epigenetic mutations similar to the mutations seen in colorectal cancer [3]. Given the rarity of these lesions and very small number of reported cases, there is no clear evidence to make a surveillance recommendation.

## 4. Conclusion

Solitary Peutz–Jeghers hamartomas are a rare occurrence but should be part of the differential diagnosis for abdominal pain from intussusception, or small bowel bleeding even in elderly patient. Balloon-assisted enteroscopy is a valuable technique for the management of solitary small bowel lesions.

## Figures and Tables

**Table 1:** All reported cases in English literature of jejunal SPJP.

Authors	Year	Country	Age	Sex	Symptoms	Diagnosed by	Definitive management	Polyp size	Underlying pathophysiology
Sone et al. [4]	2002	Japan	64 years	F	Nausea and epigastric discomfort	Upper GI endoscopy	Endoscopic electrosurgical snare polypectomy	2.8 × 2.5 × 1.8 cm	Incidental finding
Burkart et al. [5]	2007	Baltimore	35 years	F	Not specified in the article	Not specified in the article	Partial small bowel resection	NA	Intussusception
Ter Borg et al. [6]	2008	Netherlands	19 years	M	Melena	A small bowel series	Laparotomy	4 cm	GI bleeding
Pitiakoudis et al. [7]	2015	Greece	19 years	F	Abdominal pain	Abdominal ultrasound	Laparoscopy (SILS)	3 cm	Intussusception
Kalavant et al. [8]	2017	India	8 years	M	Melena	Capsule endoscopy	Laparoscopy	2 cm	Intussusception
Kalliakmanis et al. [9]	2018	Greece	16 years	F	Abdominal pain	Abdominal X-ray and then, the lesion was identified postoperatively	Laparotomy	NA	Intussusception
Suzuki et al. [10]	2020	Japan	3 months	F	Intussusception	Laparoscopic examination	Laparoscopy	3 × 3 cm	Intussusception
Endo et al. [11]	2020	Japan	29 years	M	Abdominal pain	Double Balloon endoscopy	Laparotomy (partial resection of the jejunum)	3 × 1.5 × 1 cm	Intussusception
Alenezi et al.	Index case	Quebec, Canada	81 years	M	Melena	Double-balloon enteroscopy	Endoscopic polypectomy	4 cm	GI bleeding

NA: not assigned; M: male; F: female; SILS: single-incision assisted laparoscopic surgery; numbers between brackets indicates reference.
